# Supplementary figures and images for: Dysfunctional Breathing, in COPD: A Validation Study
Source: J Clin Med. 2025 Mar 29;14(7):2353. doi: 10.3390/jcm14072353 (PMC11989668; doi:10.3390/jcm14072353)

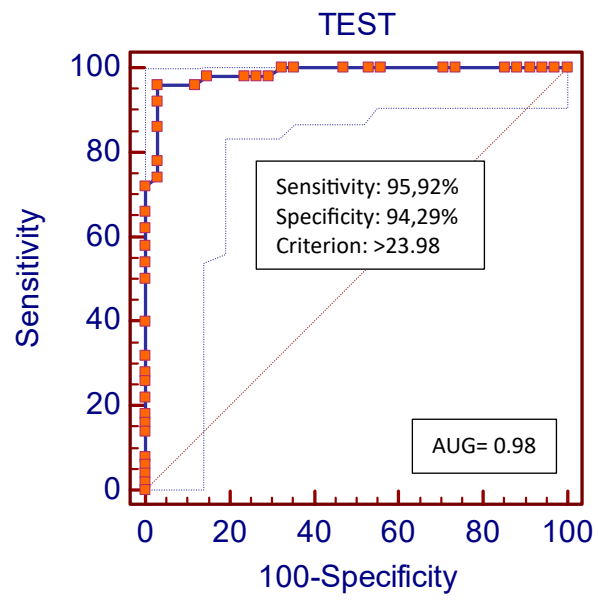

Figure S1. ROC curve for the NQ

Supplement: Supplementary file 1 [file jcm-14-02353-s001.zip › jcm-3501069-supplementary.pdf]
